# OsRbohI is the indispensable NADPH oxidase for molecular-patterns-induced reactive oxygen species production in rice

**DOI:** 10.1016/j.xplc.2024.101129

**Published:** 2024-09-12

**Authors:** Zhifang Zhao, Aiqing Sun, Wenfeng Shan, Xinhang Zheng, Ying Wang, Lu Bai, Yuchen Xu, Zhuo An, Xiaoyi Wang, Yuanmeng Wang, Jiangbo Fan

**Affiliations:** 1 Shanghai Collaborative Innovation Center of Agri-Seeds, School of Agriculture and Biology, Shanghai Jiao Tong University, Shanghai 200240, China; 2 Joint Center for Single Cell Biology, School of Agriculture and Biology, Shanghai Jiao Tong University, Shanghai 200240, China

Dear Editor,

The rapid generation of reactive oxygen species (ROS), or ROS burst, is a key feature of pathogen-associated molecular patterns (PAMPs)-triggered immunity, which is mediated by plasma membrane resident respiratory burst oxidase homologs (Rbohs) in plants (Wang et al., 2020). In *Arabidopsis*, AtRbohD is a well-documented Rboh responsible for PAMP-triggered ROS bursts, with mutations in AtRbohD leading to an abolished ROS response upon PAMP treatments. In rice, OsRbohB has been extensively studied and initially considered the counterpart to AtRbohD (Wong et al., 2007; Fan et al., 2018; Wang et al., 2020; Li et al., 2022; Wang and Kawano, 2022). However, our results indicate that mutations in *OsRbohB do not affect* ROS bursts, whereas *OsRbohI* mutants show a complete absence of ROS bursts. Consistently, *OsRbohI*, but not *OsRbohB* or *OsRbohE*, can complement the defect in *Arabidopsis AtRbohD* mutant in PAMP-triggered ROS bursts, demonstrating inter-class conservation in ROS regulation. Moreover, mutations in *OsRbohI* result in dramatically reduced resistance to rice blast. In addition, phosphorylation occurs at crucial and conserved serine sites in OsRbohI. Altogether, these results suggest that OsRbohI is the indispensable Rboh member for PAMP-induced ROS bursts in rice innate immunity.

Rice OsRbohB has long been assumed to be the major NADPH oxidase mediating PAMP-triggered ROS bursts in rice innate immunity, similar to the function of AtRbohD in *Arabidopsis* (Fan et al., 2018; Wang et al., 2020; Li et al., 2022; Wang and Kawano, 2022). However, the specific role of OsRbohB in mediating PAMP-triggered ROS bursts has not been well characterized. To investigate whether OsRbohB is involved in PAMP-triggered ROS bursts, three independent *OsRbohB* mutants were generated by CRISPR-Cas9 gene editing, targeting two specific sites (Figure 1A). Additionally, a transposon insertional *OsRbohB* mutant in the NPB background, reported as a null mutant, was obtained and characterized (Supplemental Figure 2A) (Shi et al., 2020). The ROS bursts triggered by flg22 in the four independent *OsRbohB* mutants showed no significant differences compared to the wild type (Figure 1D and 1E; Supplemental Figure 2B and 2C). These results suggest that OsRbohB may not be a major OsRboh member responsible for PAMP-triggered ROS production in rice.

**Figure 1:**
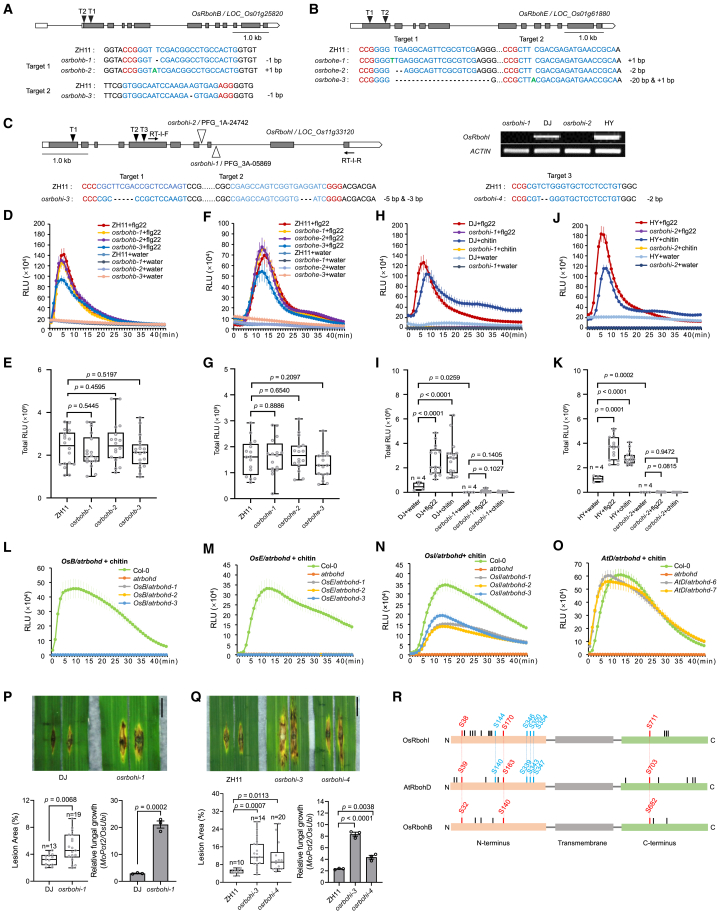
OsRbohI is the indispensable NADPH oxidase for molecular-patterns-induced reactive oxygen species (ROS) production in rice. **(A–C)** The *OsRbohB*, *OsRbohE*, and *OsRbohI* mutants generated by CRISPR-Cas9 gene editing or T-DNA insertion. Schematics of the genes are shown in the upper panels. Boxes and lines represent exons and introns, respectively. The coding regions are shaded. Black triangles indicate the target sequences of CRISPR-Cas9. The lower panel shows the sequences of CRISPR-Cas9 targets and mutations in the three independent mutants. Sequences in blue are CRISPR-Cas9 targets, and their corresponding protospacer adjacent motif sequences are in red. Dashed lines indicate deletions and bold green letters denote insertions. The two *OsRbohI* T-DNA insertion mutants are marked with transparent triangles, and *OsRbohI* transcripts in these mutants were detected by RT–PCR using the primers in **(C)**. **(D–K)** Reactive oxygen species (ROS) production induced by 100 μM flg22 or 20 μg/mL chitin in the sheath of 10-day-old rice *OsRbohB*, *OsRbohE*, and *OsRbohI* mutants and their respective wild types determined by a luminol assay. The graphs in **(D)**, **(F)**, **(H)**, and **(J)** show real-time ROS production. The columns in **(E)**, **(G)**, **(I)**, and **(K)** represent total ROS amounts, calculated from the curves shown in **(D)**, **(F)**, **(H)**, and **(J)**, respectively. Values are means ± SE, *n* = 16 (unless otherwise indicated). These experiments were conducted three times with consistent results. **(L–O)** ROS production induced by 20 μg/mL chitin in *Arabidopsis* leaf disks from different *Rboh* transgenic lines. Values are means ± SE, *n* = 18. Experiments were repeated three times with consistent results. *AtD* and *OsB*/*OsE*/*OsI* represent the *atrbohd* mutant complemented with *AtRbohD* and *OsRbohB*/*OsRbohE*/*OsRbohI*, respectively, under the *AtRbohD* promoter. **(P and Q)** Punch inoculation of rice *osrbohi* mutants and wild-type seedlings with rice blast isolate RB22. Disease symptoms were recorded at 9 and 7 days post-inoculation. Scale bar, 1 cm. Fungal biomass was determined by quantitative PCR. Values are means ± SE, *n* = 3. **(R)** Identification of phospho-sites on both termini of OsRbohI. Red labels indicate conserved phospho-sites among all three members; blue labels indicate conserved sites between OsRbohI and AtRbohD; black bars indicate species-specific phospho-sites.Statistical analyses for all data were conducted using ANOVA with Tukey’s test. In the boxplots, the center line indicates the median, box edges define the lower and upper quartiles, and whiskers denote the highest and lowest data points. All experiments were repeated at least three times, showing similar trends.

The rice genome encodes nine Rboh homologs (*OsRbohA–OsRbohI*), among which *OsRbohI*, *OsRbohB*, and *OsRbohE* are the most abundantly expressed in leaves (Supplemental Figure 1A). Six of the OsRboh members have been shown to participate in ROS generation and rice immunity (Li et al., 2022; Zhu et al., 2024). To investigate whether other OsRbohs are also involved in PAMP-triggered ROS bursts, we generated *OsRbohE* mutants using double-target gene editing and obtained three independent lines (*osrbohe-1*, *osrbohe-2*, and *osrbohe-3*) (Figure 1B). Similar to the *osrbohb* mutants, flg22-induced ROS bursts in *osrbohe* mutants showed no significant difference from those in ZH11 (Figure 1F and 1G), indicating that *OsRbohE* does not play a major role in mediating this response either.

*OsRbohI*, along with *OsRbohB* and *OsRbohE*, accounts for over 80% of the total *OsRboh* transcripts in rice leaves according to the public database (Supplemental Figure 1A). To investigate whether *OsRbohI* is involved in PAMP-triggered ROS bursts, two T-DNA insertional mutants of *OsRbohI*—*osrbohi-1*/PFG_3A-05869 in the Dongjin background and *rbohi-2*/PFG_1A-24742 in the Hwayoung background—were obtained from a rice T-DNA tagging library (Figure 1C). These T-DNA insertion mutants were confirmed as knockout mutants by RT–PCR (Figure 1C). Two additional *OsRbohI* mutants (*osrbohi-3* and *osrbohi-4*) were generated using CRISPR-Cas9 gene editing in the ZH11 background and verified by DNA sequencing (Figure 1C). These *osrbohi* mutants were subjected to ROS induction assays with flg22 and chitin. The ROS bursts were completely abolished in all four mutants (Figure 1H, 1J, 1I, and 1K; Supplemental Figure 2D–2G), suggesting that OsRbohI is an indispensable NADPH oxidase for PAMP-induced ROS bursts in rice. Notably, OsRbohI has also been implicated in mitigating asymmetric heavy metal and salinity stresses, suggesting its broader functional role beyond innate immunity (Wang et al., 2023).

OsRbohI is the closest rice Rboh to AtRbohD, sharing high sequence identity with it (Wang et al., 2023). A phylogenetic tree of the Rboh family members from rice, *Arabidopsis*, and tomato was constructed, placing OsRbohI in the same clade as AtRbohD and SlRbohB, both of which are key regulators of PAMP-induced ROS bursts (Supplemental Figure 1B) (Li et al., 2015). To determine whether *OsRbohI* is the ortholog of *AtRbohD*, the full-length coding sequences of *OsRbohI* as well as *OsRbohB*, *OsRbohE*, and *AtRbohD*, were introduced into the *Arabidopsis atrbohd* mutant under the control of the *AtRbohD* promoter to assess their abilities to complement impaired ROS production. Two to three independent lines from each transformation were subjected to ROS induction assays with flg22 or chitin. The results showed that *OsRbohI* partially restored the ROS burst defect in *atrbohd*, indicating that it retains a conserved function in ROS production, similar to *AtRbohD*, thus confirming its orthology (Figure 1L–1O and Supplemental Figure 3A–3E). To further validate its role in rice defense, *osrbohi-1*, *osrbohi-3*, and *osrbohi-4* were inoculated with blast fungus to evaluate their resistance. All three *osrbohi* mutants exhibited more severe disease symptoms and enhanced fungal growth (Figure 1P and 1Q), consistent with the role of *OsRbohI* in ROS production. Moreover, phosphorylation patterns on OsRbohI are similar to those observed for AtRbohD (described below). Altogether, these findings indicate that the mechanism of PAMP-triggered ROS production mediated by *OsRbohI and AtRbohD i*s conserved across dicotyledons and monocotyledons.

Interestingly, the *OsRbohB* and *OsRbohE* transformants did not respond to flg22 and chitin treatment despite robust expression of the transgenes, similar to mock treatments (Figure 1L and 1M; Supplemental Figure S3B, S3C, and S3F). This indicates that neither OsRbohB nor OsRbohE can catalyze ROS production during PTI, suggesting that they have evolved functions divergent from those of *AtRbohD* and *OsRbohI*. Although *OsRbohB is* not required for PAMP-induced ROS production, it may function in other stages or processes of rice immunity. This is supported by observations that all four *OsRbohB* mutants contribute to disease resistance (Supplemental Figure 4A and 4B) (Li et al., 2022; Zhu et al., 2024).

The regulation of Rbohs in plant innate immunity appears to be conserved across land plants (Chu et al., 2023). In *Arabidopsis*, AtRbohD undergoes phosphorylation by multiple kinases during PTI, including receptor-like cytoplasmic kinases BIK1/PBL1/RIPK/PBL13, calcium-dependent protein kinases CPK4/5/6/11, the MAP4 kinase SIK1, the ATP receptor DORN1, and CYSTEINE-RICH RLK2 (CRK2) (Wang et al., 2020; Wu et al., 2023). To determine whether OsRbohI is similarly regulated by phosphorylation, an immunoprecipitation-mass spectrometry (IP-MS) assay was conducted to identify phosphorylation sites on OsRbohI in rice protoplasts after chitin treatment. Phosphorylation was detected at both termini of OsRbohI, revealing seven conserved sites shared between OsRbohI and AtRbohD (Figure 1R and Supplemental Figure 5), including crucial sites S339, S343, and S347 on AtRbohD. The removal of these three phospho-sites almost completely impaired PTI-mediated, effector-triggered immunity (ETI)-mediated, and systemic acquired resistance-mediated ROS bursts (Wu et al., 2023). Similarly, phosphorylation sites on OsRbohB were also identified, though only three conserved phospho-sites were shared among the three Rboh members, excluding the crucial sites (Figure 1R and Supplemental Figure 5). The greater number of shared phospho-sites between OsRbohI and AtRbohD suggests a conserved modulation of Rboh activity, which is consistent with the inter-species complementation of the *atrbohd* mutant by *OsRbohI*. Further investigation is required to explore the regulation of OsRbohI by kinases in rice. Given that AtRbohD is involved in both PTI and ETI, it would be intriguing to investigate whether OsRbohI also functions in both PTI and ETI in rice innate immunity (Yuan et al., 2021).

## Funding

This work was supported by grants from the Shanghai Collaborative Innovation Center of Agri-Seeds (grant no. ZXWH2150201/001) and the 10.13039/501100001809Natural Science Foundation of China (grant no. 32370314) to J.F.

## Acknowledgments

We thank Fangjie Zhao (Nanjing Agricultural University), Kun-Ming Chen (Northwest A&F University), Wen-Ming Wang (Sichuan Agricultural University), Xiufang Xin (Chinese Academy of Sciences), and Yan Liang (Zhejiang University) for sharing the seeds of *osrbohi*, *osrbohb*, *atrbohd*, and *atrbohd* complementation lines, respectively. We thank Hongchun Ruan (Fujian Academy of Agricultural Sciences) for assistance with blast inoculations and Guo-Liang Wang (Ohio State University), Dongping Lu (Shanghai Jiao Tong University) and Yuese Ning (Chinese Academy of Agricultural Sciences) for their valuable comments. No conflicts of interest are declared.

## Author contributions

J.F. conceived and supervised the project. Z.Z., A.S., and W.S. designed and performed most experiments, analyzed the data, and prepared the figures. J.F. and Z.Z. wrote the manuscript. A.S., X.Z., Ying Wang, L.B., Y.X., Z.A., X.W., and Yuanmeng Wang generated the materials, related experiments, and edited the manuscript.

## Supplemental information

Document S1. Supplemental materials and methods

Document S2. Supplemental Figures 1‒5

Supplemental Table S1: Primers used in this study

Document S3. Article plus supplemental information
