# Supplementary material for: OsRbohI is the indispensable NADPH oxidase for molecular-patterns-induced reactive oxygen species production in rice
Source: Plant Commun. 2024 Sep 12;5(12):101129. doi: 10.1016/j.xplc.2024.101129 (PMC11671756; doi:10.1016/j.xplc.2024.101129)
Supplement: Document S1. Supplemental materials and methods [file mmc1.pdf]

Plant Communications, Volume *5*

Supplemental information

OsRbohI is the indispensable NADPH oxidase for molecular-patterns-induced reactive oxygen species production in rice

Zhifang Zhao, Aiqing Sun, Wenfeng Shan, Xinhang Zheng, Ying Wang, Lu Bai, Yuchen Xu, Zhuo An, Xiaoyi Wang, Yuanmeng Wang, and Jiangbo Fan

OsRbohI Is the Indispensable NADPH Oxidase for Molecular Patterns Induced Reactive Oxygen Species Production in Rice

Zhifang Zhao[1, 2, 3], Aiqing Sun[1, 2, 3], Wenfeng Shan[1, 2], Xinhang Zheng[1, 2], Ying Wang[1, 2], Yuchen Xu[1, 2], Zhuo An[1, 2], Xiaoyi Wang[1, 2], Yuanmeng Wang[1, 2], Jiangbo Fan[1, 2, *]

1 Shanghai Collaborative Innovation Center of Agri-Seeds, School of Agriculture and Biology, Shanghai Jiao Tong University

2 Joint Center for Single Cell Biology, School of Agriculture and Biology, Shanghai Jiao Tong University

[3] These authors contributed equally.

* Correspondence: Jiangbo Fan (fan.jb@sjtu.edu.cn)

Materials and methods

Plant Materials and Growth Conditions

Mutants of *OsRbohI* (*LOC_Os11g33120), OsRbohB* (*LOC_Os01g25820*) and *OsRbohE* (*LOC_Os01g61880*) in the Zhonghua 11 (ZH11) background were generated using CRISPR/Cas9. The CRISPR targets of them were selected with online tool (http://crispr.mit.edu/). The CRISPR constructs were transformed into rice by service agent (WIMI Biotech) and confirmed by DNA sequencing. T-DNA insertion mutants of *OsRbohI* were obtained from rice T-DNA tagging library (POSTECH) under accession numbers PFG_3A-05869 (*osrbohi-1*) and PFG_1A-24742 (*osrbohi-2*). Mutant *osrbohi-3* is shared by Prof. Fangjie Zhao (Nanjing Agricultural University) as reported (Wang et al., 2023). The mutant lines *osrbohb-1* and *osrbohb-2* are kind gifts from Prof. Yuese Ning (Chinese Academy of Agricultural Sciences). Mutants *osrbohb-3* and *osrbohi-4* are obtained from BIOGLE GeneTech. Rice mutant *osrbohb-4* is a Tos17 insertional line (accession ID: NF5029_0_501_1A) gifted by Prof. Kun-Ming Chen (Northwest A&F University) as reported (Shi et al., 2020). Arabidopsis mutant

atrbohd is shared by Prof. Xiufang Xin (Chinese Academy of Sciences) as reported (Yuan et al., 2021). The *atrbohd* complementary lines *AtD-6* and *AtD-7* are gifted by Yan Liang (Zhejiang University) as reported (Qi et al., 2024). Rice plants were grown in a growth room under the conditions of 12-h day, 30℃, 80% relative humidity (RH) followed by 12-h dark, 28℃, 60% RH. Arabidopsis plants were grown in a growth room at 22 °C with a 16-h-light and 8-h-dark cycle with 65% RH.

Arabidopsis transformation

Full-length CDS of *OsRbohB*/*E*/*I* and *AtRbohD* were inserted downstream the native promoter of *AtRbohD* (2041 bp upstream start codon) and then introduced into *Agrobacterium* strain GV3101. All the Arabidopsis transformation were conducted using floral dip method as previously described (Sun et al., 2022). Hygromycin was used to select transformed lines.

qRT-PCR and RT-PCR

To detect the transcripts of *OsRbohB*, *OsRbohE, OsRbohI* and *AtRbohD* in different mutants and corresponding WT, leaves from 10-day-old seedlings grown on 1/2 MS medium in sterile glass vessel (for rice) or culture dish (for Arabidopsis) were ground in liquid nitrogen. Total RNAs were extracted with TaKaRa MiniBEST Universal RNA Extraction Kit. First-strand cDNA synthesis was conducted with ReverTra Ace qPCR RT Master Mix with gDNA Remover (TOYOBO). For *osrbohi-1* and *osrbohi-2*, RT-PCRs were performed with primers spanning T-DNA insertion with *OsACTIN* as an internal control. For Arabidopsis, *AtActin2* was used as an internal control. Primers used are shown in Supplemental Table S1.

ROS measurement

Detection of PAMP triggered ROS were conducted with a chemiluminescence assay as previously described (Wang et al., 2022). The rice sheaths or Arabidopsis leaf discs were treated with 100 μM flg22 (Sangon Biotech), 20 μg/mL chitin (Sigma), or sterile water in 50 mM Tris-HCl buffer (pH 7.5), containing 10 μM L-012 (FUJIFILM) and 10 μg/mL horseradish peroxidase (Sigma). Luminescence was monitored immediately after the treatment and continuously measured at 1-min intervals for 30-45 minutes with Varioskan Flash multireader (BioTek).

Rice Blast Inoculations and Disease Evaluations

Blast fungal punch inoculation of rice was conducted as previously described (Fan et al., 2018) with modifications. *M. oryzae* (isolate RB22) was cultured on oatmeal agar at 25℃ for 10 days to produce spores. Fresh spores were then collected with distilled H_2O containing 0.5% Tween-20 and adjusted to a concentration of 5×10^5 spores/ml. Seedlings were cultured in big pots (16 plants per pot) for six weeks. The middle part of second leaf (count from top) of each plant was punched and the phenotype was scored at 7~12 dpi. The representative leaves of each line were collected and gDNA were extracted using Cetyltrimethylammonium Bromide (CTAB). The fungal biomass was determined by quantifying the fungal transposon *MoPot2* DNA with quantitative PCR, normalized to rice ubiquitin gene (*LOC_Os03 g13170*) (Kawano et al., 2010). PCR primers are listed in Supplemental Table S1.

Immunoprecipitation (IP) and mass spectrometry assay

IP-MS assay was conducted with rice protoplasts as previously described (Fan et al., 2018). Wild type rice seedlings of 10-14-day old cultured under lights on ½ MS medium were used for protoplast isolation. Briefly, OsRbohB and OsRbohI with HA or GFP label were transformed into rice protoplast by PEG-$CaCl_2$. The resulted protoplasts were treated with 20 μg/ml chitin and then IP with HA or GFP beads for mass spectrometry to identify protein phosphorylation sites.

REFERENCE

Fan, J., Bai, P., Ning, Y., Wang, J., Shi, X., Xiong, Y., Zhang, K., He, F., Zhang, C., Wang, R., *et al.* (2018). The Monocot-Specific Receptor-like Kinase SDS2 Controls Cell Death and Immunity in Rice. Cell Host Microbe *23*, 498-510 e495.

Kawano, Y., Akamatsu, A., Hayashi, K., Housen, Y., Okuda, J., Yao, A., Nakashima, A., Takahashi, H., Yoshida, H., Wong, H.L., *et al.* (2010). Activation of a Rac GTPase by the NLR Family Disease Resistance Protein Pit Plays a Critical Role in Rice Innate Immunity. Cell Host & Microbe *7*, 362-375.

Qi, F., Li, J., Ai, Y., Shangguan, K., Li, P., Lin, F., and Liang, Y. (2024). DGK5β-derived phosphatidic acid regulates ROS production in plant immunity by stabilizing NADPH oxidase. Cell Host & Microbe *32*, 425-440.e427.

Shi, Y., Chang, Y.L., Wu, H.T., Shalmani, A., Liu, W.T., Li, W.Q., Xu, J.W., and Chen, K.M. (2020). OsRbohB-mediated ROS production plays a crucial role in drought stress tolerance of rice. Plant Cell Rep *39*, 1767-1784.

Sun, A., Yin, C., Ma, M., Zhou, Y., Zheng, X., Tu, X., and Fang, Y. (2022). Feedback regulation of auxin signaling through the transcription of H2A.Z and deposition of H2A.Z to SMALL AUXIN UP RNAs in Arabidopsis. New Phytol *236*(5), 1721-1733.

Wang, H.-Q., Zhao, X.-Y., Xuan, W., Wang, P., and Zhao, F.-J. (2023). Rice roots avoid asymmetric heavy metal and salinity stress via an RBOH-ROS-auxin signaling cascade. Molecular Plant *16*, 1678-1694.

Wang, Y., An, Z., Zhao, Z., Li, C., and Fan, J. (2022). Real-Time Detection of Reactive Oxygen Species Production in Immune Response in Rice with a Chemiluminescence Assay. Journal of Visualized Experiments *189*, e64776.

Yuan, M., Jiang, Z., Bi, G., Nomura, K., Liu, M., Wang, Y., Cai, B., Zhou, J.M., He, S.Y., and Xin, X.F. (2021). Pattern-recognition receptors are required for NLR-mediated plant immunity. Nature *592*(7852), 105-109..
