# Supplementary material for: OsRbohI is the indispensable NADPH oxidase for molecular-patterns-induced reactive oxygen species production in rice
Source: Plant Commun. 2024 Sep 12;5(12):101129. doi: 10.1016/j.xplc.2024.101129 (PMC11671756; doi:10.1016/j.xplc.2024.101129)
Supplement: Document S2. Supplemental Figures 1‒5 [file mmc2.pdf]

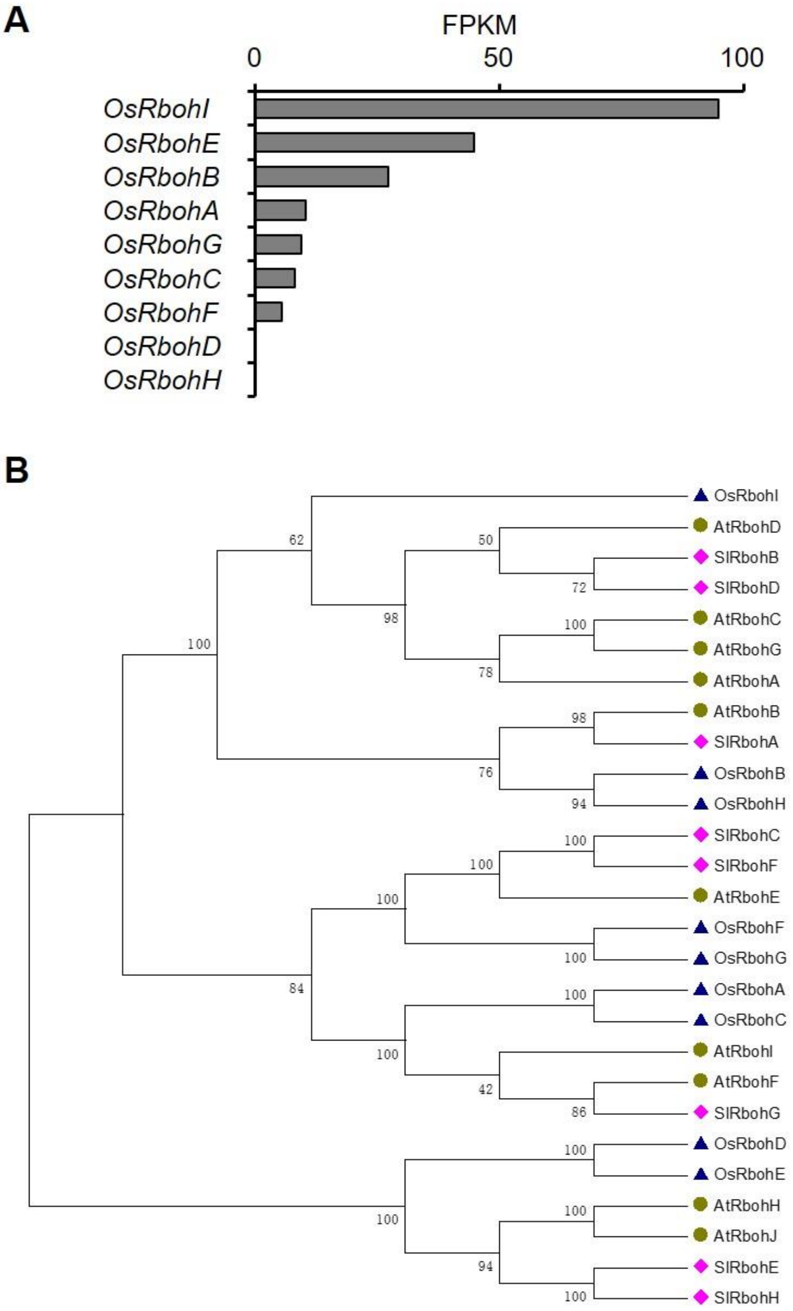

Supplemental Figure 1. Expression pattern and phylogenetic relationships of OsRbohs. (A) Expression of 9 *OsRboh* genes in leaves at about 3 weeks. Data are obtained from public database (Bidzinski *et al.*, 2016). FPKM: Fragments Per Kilobase of exon model per Million mapped fragments. (B) Phylogenetic analysis of RBOH family proteins in *Oryza sativa* (*Os*), *Arabidopsis thaliana* (At), and *Solanum lycopersicum* (*Sl*). Full length proteins were used to construct the tree. Numbers at the nodes indicate the bootstrap values on neighbor joining analysis.

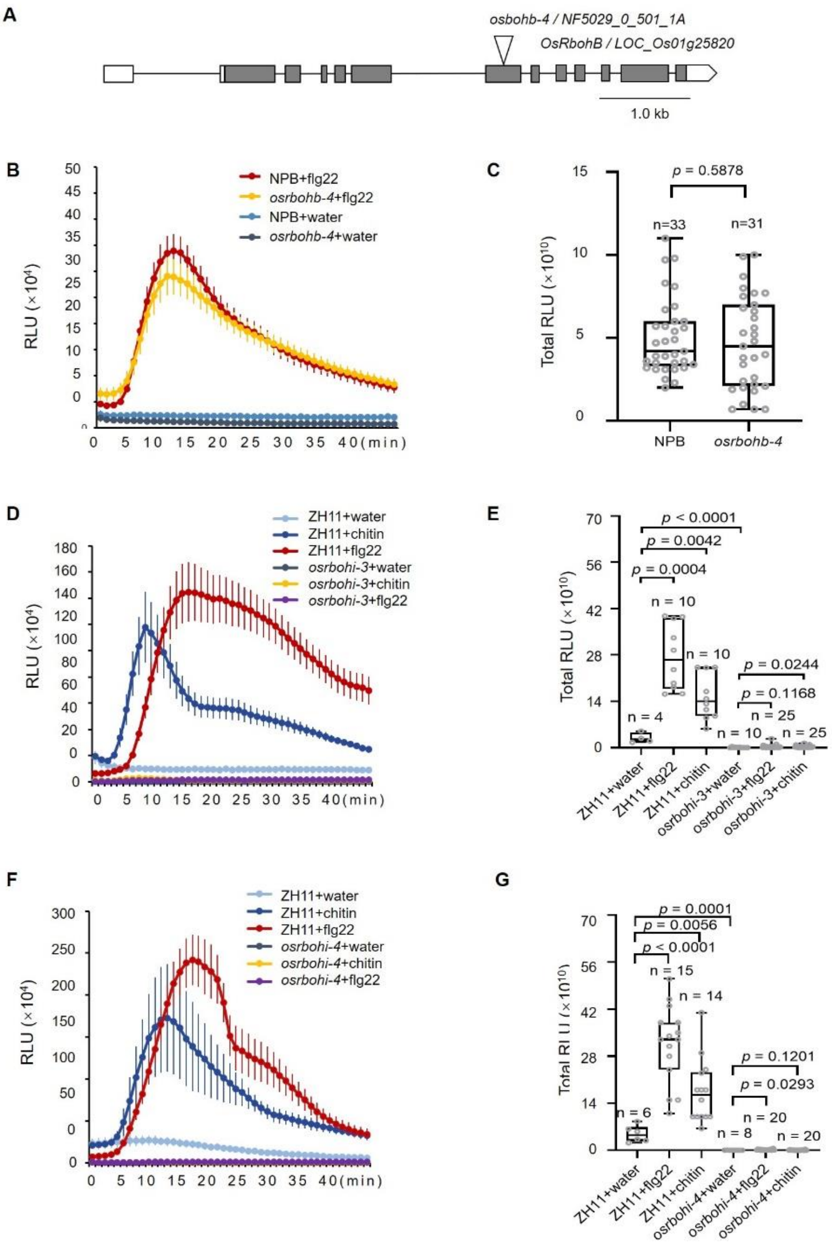

Supplemental Figure 2. PAMP-induced ROS production was unaltered in *OsRbohB* while totally abolished in *OsRbohI* mutants. (A) Schema of *Tos17* insertional mutant *osrbohb-4*. Boxes and lines represent exons and introns respectively. Transparent triangles mark the T-DNA insertion. (B) (D) and (F) ROS production induced by flg22 or chitin on 10-day-old rice sheath of *OsRboh* and corresponding WT. Values are means ± SE. (C) (E) and (G) The total amount of ROS generated with the rice sheath calculated from the curve corresponding to (B) (D) and (F), respectively. Experiments were performed 3 times with similar results. Statistical analysis was conducted by ANOVA with Tukey's test.

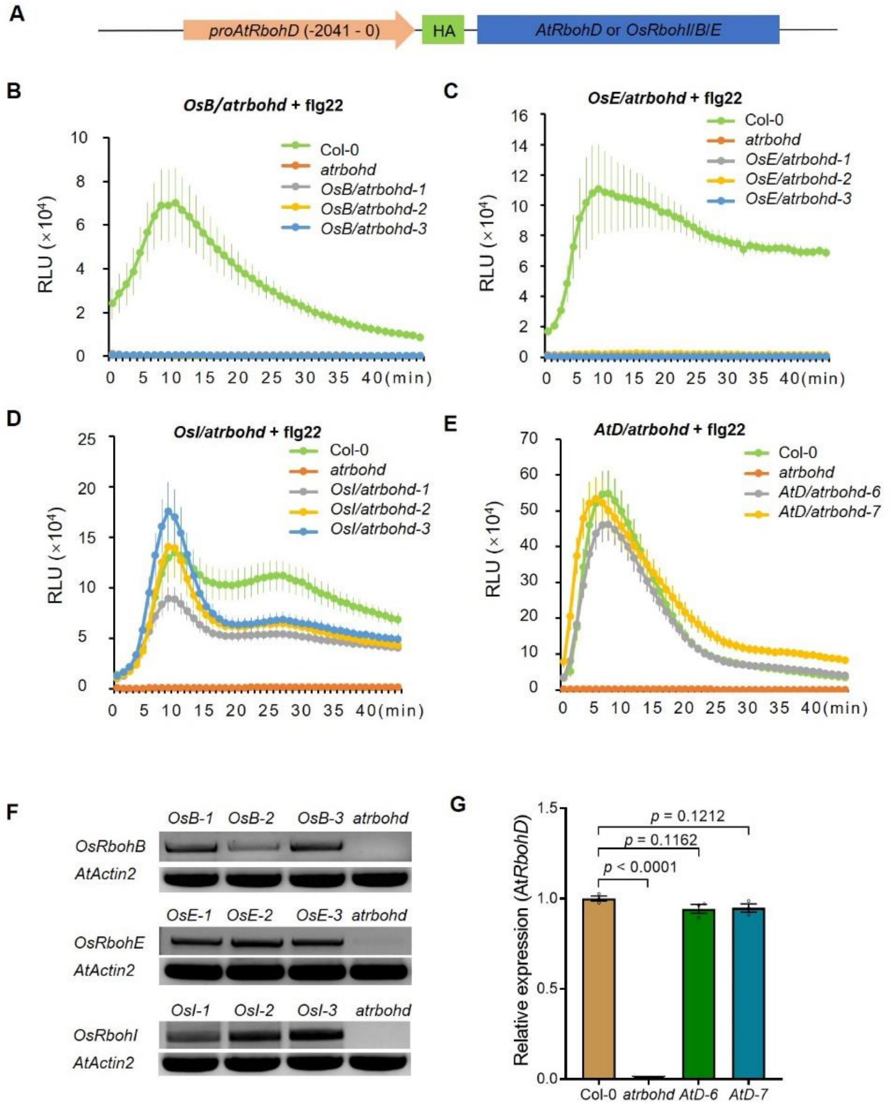

Supplemental Figure 3. ***OsRbohI,* but not *OsRbohB* or *OsRbohE*, rescues the defect of Arabidopsis *atrbohd* mutant in PAMP-triggered ROS burst.** (A) A schematic explanation for vectors construction in complementation studies. (B) -(E) ROS production induced by 1 μM flg22 on Arabidopsis leaf discs of different *Rboh* transgenic lines. Values are means ± SE, n = 18. (F) Transcripts of *OsRbohB/E/I* in WT(Col-0), *atrbohd* and complementary lines were detected by RT-PCR. (G) Transcripts of *AtRbohD* in WT(Col-0), *atrbohd* and complementary lines were quantified by qRT-PCR. Experiments were performed 3 times with similar results. *AtD* and *OsB*/*E*/*I* indicate *atrbohd* mutant complemented with *AtRbohD* and *OsRbohB*/*E*/*I*, respectively. Data presented are the means ± SE from three replicates. Statistical analysis was conducted by ANOVA with Tukey’s test.

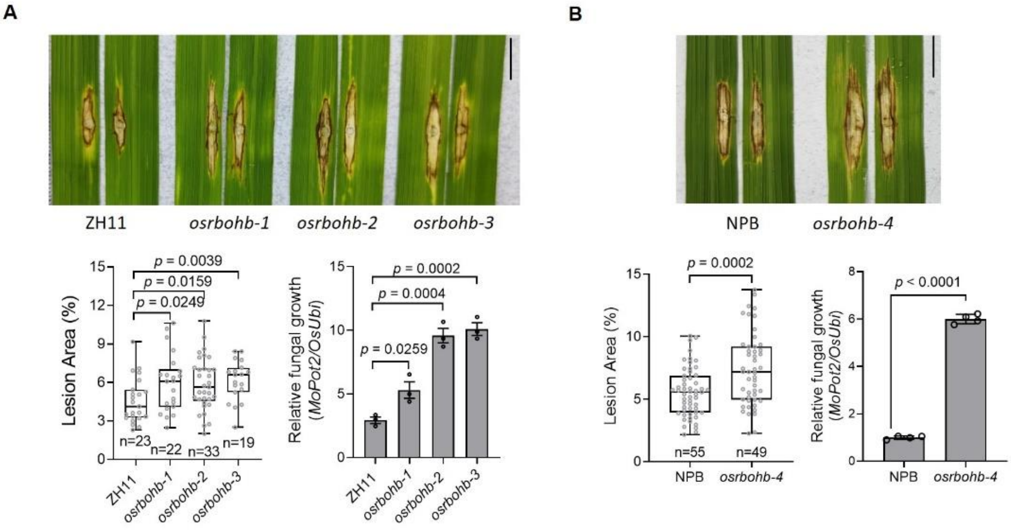

Supplemental Figure 4. **Assay of blast disease resistance on *osrbohb* mutants.** (A) and (B) Punch inoculation of *osrbohb* mutants and wild-type seedlings with rice blast isolate RB22. The disease symptom was recorded 12 days post-inoculation (dpi). Bar = 1 cm. Experiments were performed 3 times with similar results. Statistical analysis was conducted by ANOVA with Tukey's test.

Sample	Phospho-sites	MS Coverage
OsRbohI-GFP-1	S38, S42, S58, S62, S101, S125, S127, S144, S354, S711, S792, S793	69%
OsRbohI-GFP-2	S38, S56, S58, S62, S126, S127, S144, S346, S350, S354, S711, S790, S792, S793	71%
HA-OsRbohI-1	S38, S58, S65, S127, S170, S350, S354, S711	57%
HA-OsRbohI-2	S38, S58, S62, S125, S127, S144, S170, S350, S354	60%
OsRbohB-GFP-1	S32, S78, S91, S140, S682, S690, S750	58%
OsRbohB-GFP-2	S32, S78, S91, S121, S140, S682, S750	57%

Supplemental Figure 5. **Assay of phosphorylation sites on OsRbohI and OsRbohB.** Phosphorylation sites on OsRbohI and OsRbohB as determined by IP-MS. OsRbohI and OsRbohB tagged with GFP at C-terminal (or HA at N-terminal) were expressed in rice protoplast, treated with chitin and applied to IP-MS analysis.
