# Supplementary material for: OsRbohI is the indispensable NADPH oxidase for molecular-patterns-induced reactive oxygen species production in rice
Source: Plant Commun. 2024 Sep 12;5(12):101129. doi: 10.1016/j.xplc.2024.101129 (PMC11671756; doi:10.1016/j.xplc.2024.101129)
Supplement: Document S3. Article plus supplemental information [file mmc4.pdf]

Plant Communications
Correspondence

CellPress
Partner Journal

OsRbohI is the indispensable NADPH oxidase for molecular-patterns-induced reactive oxygen species production in rice

Dear Editor,

The rapid generation of reactive oxygen species (ROS), or ROS burst, is a key feature of pathogen-associated molecular patterns (PAMPs)-triggered immunity, which is mediated by plasma membrane resident respiratory burst oxidase homologs (Rbohs) in plants (Wang et al., 2020). In *Arabidopsis*, AtRbohD is a well-documented Rboh responsible for PAMP-triggered ROS bursts, with mutations in AtRbohD leading to an abolished ROS response upon PAMP treatments. In rice, OsRbohB has been extensively studied and initially considered the counterpart to AtRbohD (Wong et al., 2007; Fan et al., 2018; Wang et al., 2020; Li et al., 2022; Wang and Kawano, 2022). However, our results indicate that mutations in *OsRbohB do not affect* ROS bursts, whereas *OsRbohI* mutants show a complete absence of ROS bursts. Consistently, *OsRbohI*, but not *OsRbohB* or *OsRbohE*, can complement the defect in *Arabidopsis AtRbohD* mutant in PAMP-triggered ROS bursts, demonstrating inter-class conservation in ROS regulation. Moreover, mutations in *OsRbohI* result in dramatically reduced resistance to rice blast. In addition, phosphorylation occurs at crucial and conserved serine sites in OsRbohI. Altogether, these results suggest that OsRbohI is the indispensable Rboh member for PAMP-induced ROS bursts in rice innate immunity.

Rice OsRbohB has long been assumed to be the major NADPH oxidase mediating PAMP-triggered ROS bursts in rice innate immunity, similar to the function of AtRbohD in *Arabidopsis* (Fan et al., 2018; Wang et al., 2020; Li et al., 2022; Wang and Kawano, 2022). However, the specific role of OsRbohB in mediating PAMP-triggered ROS bursts has not been well characterized. To investigate whether OsRbohB is involved in PAMP-triggered ROS bursts, three independent *OsRbohB* mutants were generated by CRISPR-Cas9 gene editing, targeting two specific sites (Figure 1A). Additionally, a transposon insertional *OsRbohB* mutant in the NPB background, reported as a null mutant, was obtained and characterized (Supplemental Figure 2A) (Shi et al., 2020). The ROS bursts triggered by flg22 in the four independent *OsRbohB* mutants showed no significant differences compared to the wild type (Figure 1D and 1E; Supplemental Figure 2B and 2C). These results suggest that OsRbohB may not be a major OsRboh member responsible for PAMP-triggered ROS production in rice.

The rice genome encodes nine Rboh homologs (*OsRbohA–OsRbohI*), among which *OsRbohI*, *OsRbohB*, and *OsRbohE* are the most abundantly expressed in leaves (Supplemental Figure 1A). Six of the OsRboh members have been shown to participate in ROS generation and rice immunity (Li et al., 2022; Zhu et al., 2024). To investigate whether other OsRbohs are also involved in PAMP-triggered ROS bursts, we generated *OsRbohE* mutants using double-target gene editing and obtained three independent lines (*osrbohe-1*, *osrbohe-2*, and *osrbohe-3*) (Figure 1B). Similar to the *osrbohb* mutants, flg22-induced ROS bursts in *osrbohe* mutants showed no significant difference from those in ZH11 (Figure 1F and 1G), indicating that *OsRbohE* does not play a major role in mediating this response either.

OsRbohI, along with *OsRbohB* and *OsRbohE*, accounts for over 80% of the total *OsRboh* transcripts in rice leaves according to the public database (Supplemental Figure 1A). To investigate whether *OsRbohI* is involved in PAMP-triggered ROS bursts, two T-DNA insertional mutants of *OsRbohI*—*osrbohi-1*/PFG_3A-05869 in the Dongjin background and *rbohi-2*/PFG_1A-24742 in the Hwayoung background—were obtained from a rice T-DNA tagging library (Figure 1C). These T-DNA insertion mutants were confirmed as knockout mutants by RT–PCR (Figure 1C). Two additional *OsRbohI* mutants (*osrbohi-3* and *osrbohi-4*) were generated using CRISPR-Cas9 gene editing in the ZH11 background and verified by DNA sequencing (Figure 1C). These *osrbohi* mutants were subjected to ROS induction assays with flg22 and chitin. The ROS bursts were completely abolished in all four mutants (Figure 1H, 1J, 1I, and 1K; Supplemental Figure 2D–2G), suggesting that OsRbohI is an indispensable NADPH oxidase for PAMP-induced ROS bursts in rice. Notably, OsRbohI has also been implicated in mitigating asymmetric heavy metal and salinity stresses, suggesting its broader functional role beyond innate immunity (Wang et al., 2023).

OsRbohI is the closest rice Rboh to AtRbohD, sharing high sequence identity with it (Wang et al., 2023). A phylogenetic tree of the Rboh family members from rice, *Arabidopsis*, and tomato was constructed, placing OsRbohI in the same clade as AtRbohD and SlRbohB, both of which are key regulators of PAMP-induced ROS bursts (Supplemental Figure 1B) (Li et al., 2015). To determine whether *OsRbohI* is the ortholog of *AtRbohD*, the full-length coding sequences of *OsRbohI* as well as *OsRbohB*, *OsRbohE*, and *AtRbohD*, were introduced into the *Arabidopsis atrbohd* mutant under the control of the *AtRbohD* promoter to assess their abilities to complement impaired ROS production. Two to three independent lines from each transformation were subjected to ROS induction assays with flg22 or chitin. The results showed that *OsRbohI* partially restored the ROS burst defect in *atrbohd*, indicating that it retains a conserved function in ROS production, similar to

Published by the Plant Communications Shanghai Editorial Office in association with Cell Press, an imprint of Elsevier Inc., on behalf of CSPB and CEMPS, CAS.

This is an open access article under the CC BY license (http://creativecommons.org/licenses/by/4.0/).

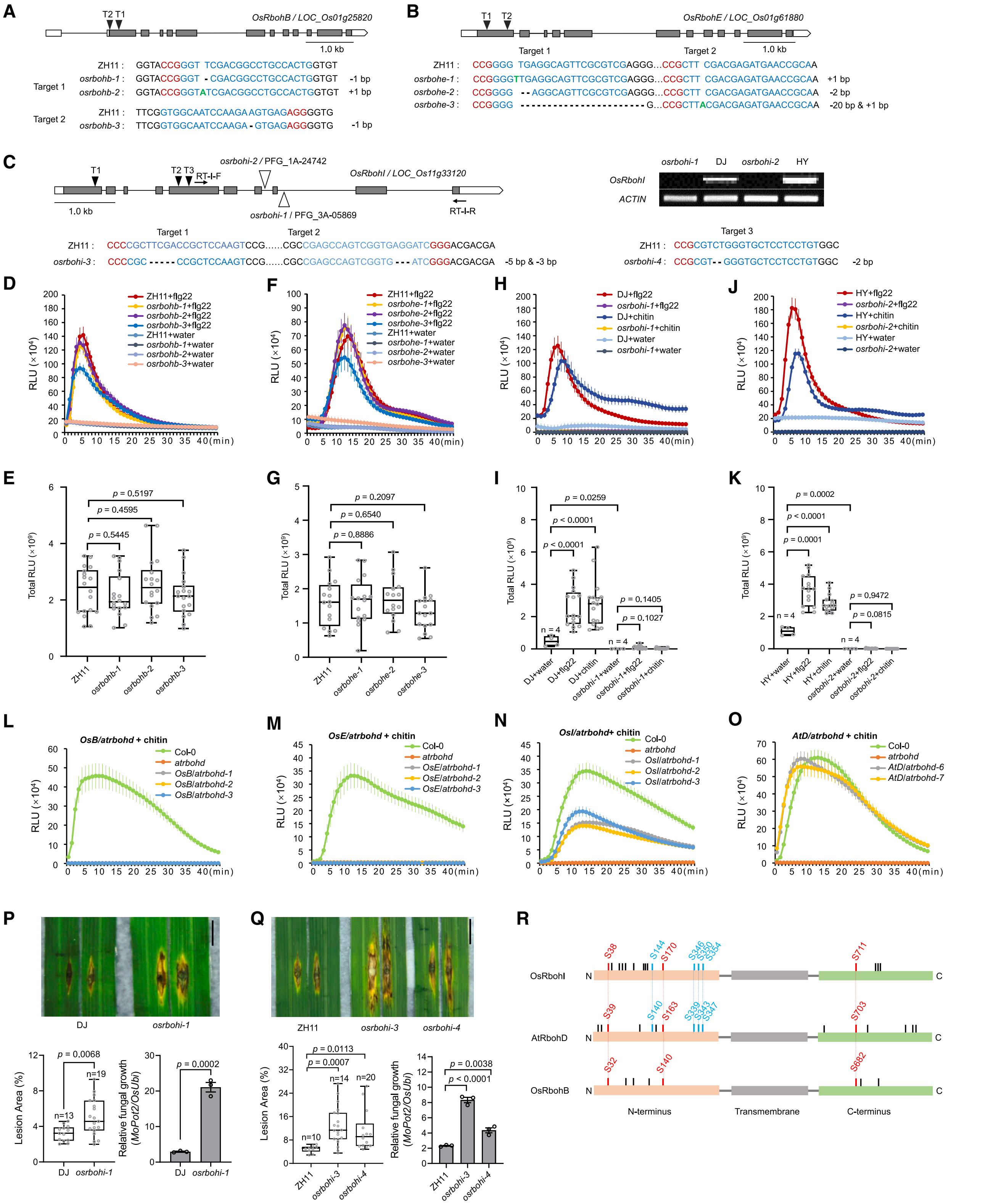

Figure 1. OsRbohI is the indispensable NADPH oxidase for molecular-patterns-induced reactive oxygen species (ROS) production in rice.

(A–C) The *OsRbohB*, *OsRbohE*, and *OsRbohI* mutants generated by CRISPR-Cas9 gene editing or T-DNA insertion. Schematics of the genes are shown in the upper panels. Boxes and lines represent exons and introns, respectively. The coding regions are shaded. Black triangles indicate the target

(legend continued on next page)

AtRbohD, thus confirming its orthology (Figure 1L–1O and Supplemental Figure 3A–3E). To further validate its role in rice defense, *osrbohi-1*, *osrbohi-3*, and *osrbohi-4* were inoculated with blast fungus to evaluate their resistance. All three *osrbohi* mutants exhibited more severe disease symptoms and enhanced fungal growth (Figure 1P and 1Q), consistent with the role of *OsRbohI* in ROS production. Moreover, phosphorylation patterns on OsRbohI are similar to those observed for AtRbohD (described below). Altogether, these findings indicate that the mechanism of PAMP-triggered ROS production mediated by *OsRbohI and AtRbohD i*s conserved across dicotyledons and monocotyledons.

Interestingly, the *OsRbohB* and *OsRbohE* transformants did not respond to flg22 and chitin treatment despite robust expression of the transgenes, similar to mock treatments (Figure 1L and 1M; Supplemental Figure S3B, S3C, and S3F). This indicates that neither OsRbohB nor OsRbohE can catalyze ROS production during PTI, suggesting that they have evolved functions divergent from those of *AtRbohD* and *OsRbohI*. Although *OsRbohB is* not required for PAMP-induced ROS production, it may function in other stages or processes of rice immunity. This is supported by observations that all four *OsRbohB* mutants contribute to disease resistance (Supplemental Figure 4A and 4B) (Li et al., 2022; Zhu et al., 2024).

The regulation of Rbohs in plant innate immunity appears to be conserved across land plants (Chu et al., 2023). In *Arabidopsis*, AtRbohD undergoes phosphorylation by multiple kinases during PTI, including receptor-like cytoplasmic kinases BIK1/PBL1/RIPK/PBL13, calcium-dependent protein kinases CPK4/5/6/11, the MAP4 kinase SIK1, the ATP receptor DORN1, and CYSTEINE-RICH RLK2 (CRK2) (Wang et al., 2020; Wu et al., 2023). To determine whether OsRbohI is similarly regulated by phosphorylation, an immunoprecipitation-mass spectrometry (IP-MS) assay was conducted to identify phosphorylation sites on OsRbohI in rice protoplasts after chitin treatment. Phosphorylation was detected at both termini of OsRbohI, revealing seven conserved sites shared between OsRbohI and AtRbohD (Figure 1R and Supplemental Figure 5), including crucial sites S339, S343, and S347 on AtRbohD. The removal of these three phospho-sites almost completely impaired PTI-mediated, effector-triggered immunity (ETI)-mediated, and systemic acquired resistance-mediated ROS bursts (Wu et al., 2023). Similarly, phosphorylation sites on OsRbohB were also identified, though only three conserved phospho-sites were shared among the three Rboh members, excluding the crucial sites (Figure 1R and Supplemental Figure 5). The greater number of shared phospho-sites between OsRbohI and AtRbohD suggests a conserved modulation of Rboh activity, which is consistent with the inter-species complementation of the *atrbohd* mutant by *OsRbohI*. Further investigation is required to explore the regulation of OsRbohI by kinases in rice. Given that AtRbohD is involved in both PTI and ETI, it would be intriguing to investigate whether OsRbohI also functions in both PTI and ETI in rice innate immunity (Yuan et al., 2021).

FUNDING

This work was supported by grants from the Shanghai Collaborative Innovation Center of Agri-Seeds (grant no. ZXWH2150201/001) and the Natural Science Foundation of China (grant no. 32370314) to J.F.

ACKNOWLEDGMENTS

We thank Fangjie Zhao (Nanjing Agricultural University), Kun-Ming Chen (Northwest A&F University), Wen-Ming Wang (Sichuan Agricultural University), Xiufang Xin (Chinese Academy of Sciences), and Yan Liang (Zhejiang University) for sharing the seeds of *osrbohi*, *osrbohb*, *atrbohd*, and *atrbohd* complementation lines, respectively. We thank Hongchun Ruan (Fujian Academy of Agricultural Sciences) for assistance with blast inoculations and Guo-Liang Wang (Ohio State University), Dongping Lu (Shanghai Jiao Tong University) and Yuese Ning (Chinese Academy of Agricultural Sciences) for their valuable comments. No conflicts of interest are declared.

AUTHOR CONTRIBUTIONS

J.F. conceived and supervised the project. Z.Z., A.S., and W.S. designed and performed most experiments, analyzed the data, and prepared the figures. J.F. and Z.Z. wrote the manuscript. A.S., X.Z., Ying Wang, L.B., Y.X., Z.A., X.W., and Yuanmeng Wang generated the materials, related experiments, and edited the manuscript.

SUPPLEMENTAL INFORMATION

Supplemental information is available at *Plant Communications Online*.

Received: April 18, 2024
Revised: July 31, 2024
Accepted: September 5, 2024
Published: September 12, 2024

sequences of CRISPR-Cas9. The lower panel shows the sequences of CRISPR-Cas9 targets and mutations in the three independent mutants. Sequences in blue are CRISPR-Cas9 targets, and their corresponding protospacer adjacent motif sequences are in red. Dashed lines indicate deletions and bold green letters denote insertions. The two *OsRbohI* T-DNA insertion mutants are marked with transparent triangles, and *OsRbohI* transcripts in these mutants were detected by RT–PCR using the primers in **(C)**.

(D–K) Reactive oxygen species (ROS) production induced by 100 μM flg22 or 20 μg/mL chitin in the sheath of 10-day-old rice *OsRbohB*, *OsRbohE*, and *OsRbohI* mutants and their respective wild types determined by a luminol assay. The graphs in **(D)**, **(F)**, **(H)**, and **(J)** show real-time ROS production. The columns in **(E)**, **(G)**, **(I)**, and **(K)** represent total ROS amounts, calculated from the curves shown in **(D)**, **(F)**, **(H)**, and **(J)**, respectively. Values are means ± SE, $n = 16$ (unless otherwise indicated). These experiments were conducted three times with consistent results.

(L–O) ROS production induced by 20 μg/mL chitin in *Arabidopsis* leaf disks from different *Rboh* transgenic lines. Values are means ± SE, $n = 18$. Experiments were repeated three times with consistent results. *AtD* and *OsB*/*OsE*/*OsI* represent the *atrbohd* mutant complemented with *AtRbohD* and *OsRbohB*/*OsRbohE*/*OsRbohI*, respectively, under the *AtRbohD* promoter.

(P and Q) Punch inoculation of rice *osrbohi* mutants and wild-type seedlings with rice blast isolate RB22. Disease symptoms were recorded at 9 and 7 days post-inoculation. Scale bar, 1 cm. Fungal biomass was determined by quantitative PCR. Values are means ± SE, $n = 3$.

(R) Identification of phospho-sites on both termini of OsRbohI. Red labels indicate conserved phospho-sites among all three members; blue labels indicate conserved sites between OsRbohI and AtRbohD; black bars indicate species-specific phospho-sites.Statistical analyses for all data were conducted using ANOVA with Tukey's test. In the boxplots, the center line indicates the median, box edges define the lower and upper quartiles, and whiskers denote the highest and lowest data points. All experiments were repeated at least three times, showing similar trends.

Zhifang Zhao[1,2,3], Aiqing Sun[1,2,3], Wenfeng Shan[1,2], Xinhang Zheng[1,2], Ying Wang[1,2], Lu Bai[1,2], Yuchen Xu[1,2], Zhuo An[1,2], Xiaoyi Wang[1,2], Yuanmeng Wang[1,2] and Jiangbo Fan[1,2,*]

[1]Shanghai Collaborative Innovation Center of Agri-Seeds, School of Agriculture and Biology, Shanghai Jiao Tong University, Shanghai 200240, China
[2]Joint Center for Single Cell Biology, School of Agriculture and Biology, Shanghai Jiao Tong University, Shanghai 200240, China
[3]These authors contributed equally to this article.
***Correspondence: Jiangbo Fan (fan.jb@sjtu.edu.cn)**
https://doi.org/10.1016/j.xplc.2024.101129

REFERENCES

Chu, J., Monte, I., DeFalco, T.A., Köster, P., Derbyshire, P., Menke, F.L.H., and Zipfel, C. (2023). Conservation of the PBL-RBOH immune module in land plants. Curr. Biol. **33**:1130–1137.e5.

Fan, J., Bai, P., Ning, Y., Wang, J., Shi, X., Xiong, Y., Zhang, K., He, F., Zhang, C., Wang, R., et al. (2018). The Monocot-Specific Receptor-like Kinase SDS2 Controls Cell Death and Immunity in Rice. Cell Host Microbe **23**:498–510.e5.

Li, G.B., He, J.X., Wu, J.L., Wang, H., Zhang, X., Liu, J., Hu, X.H., Zhu, Y., Shen, S., Bai, Y.F., et al. (2022). Overproduction of OsRACK1A, an effector-targeted scaffold protein promoting OsRBOHB-mediated ROS production, confers rice floral resistance to false smut disease without yield penalty. Mol. Plant **15**:1790–1806.

Li, X., Zhang, H., Tian, L., Huang, L., Liu, S., Li, D., and Song, F. (2015). Tomato SlRbohB, a member of the NADPH oxidase family, is required for disease resistance against Botrytis cinerea and tolerance to drought stress. Front. Plant Sci. **6**:463.

Shi, Y., Chang, Y.L., Wu, H.T., Shalmani, A., Liu, W.T., Li, W.Q., Xu, J.W., and Chen, K.M. (2020). OsRbohB-mediated ROS production plays a crucial role in drought stress tolerance of rice. Plant Cell Rep. **39**:1767–1784.

Wang, H.-Q., Zhao, X.-Y., Xuan, W., Wang, P., and Zhao, F.-J. (2023). Rice roots avoid asymmetric heavy metal and salinity stress via an RBOH-ROS-auxin signaling cascade. Mol. Plant **16**:1678–1694.

Wang, Q., and Kawano, Y. (2022). Improving disease resistance to rice false smut without yield penalty by manipulating the expression of effector target. Mol. Plant **15**:1834–1837.

Wang, R., He, F., Ning, Y., and Wang, G.L. (2020). Fine-Tuning of RBOH-Mediated ROS Signaling in Plant Immunity. Trends Plant Sci. **25**:1060–1062.

Wong, H.L., Pinontoan, R., Hayashi, K., Tabata, R., Yaeno, T., Hasegawa, K., Kojima, C., Yoshioka, H., Iba, K., Kawasaki, T., and Shimamoto, K. (2007). Regulation of rice NADPH oxidase by binding of Rac GTPase to its N-terminal extension. Plant Cell **19**:4022–4034.

Wu, B., Qi, F., and Liang, Y. (2023). Fuels for ROS signaling in plant immunity. Trends Plant Sci. **28**:1124–1131.

Yuan, M., Ngou, B.P.M., Ding, P., and Xin, X.-F. (2021). PTI-ETI crosstalk: an integrative view of plant immunity. Curr. Opin. Plant Biol. **62**:102030.

Zhu, Y., Su, H., Liu, X.-X., Sun, J.-F., Xiang, L., Liu, Y.-J., Hu, Z.-W., Xiong, X.-Y., Yang, X.-M., Bhutto, S.H., et al. (2024). Identification of NADPH Oxidase Genes Crucial for Rice Multiple Disease Resistance and Yield Traits. Rice **17**:1.

Plant Communications, Volume *5*

Supplemental information

OsRbohI is the indispensable NADPH oxidase for molecular-patterns-induced reactive oxygen species production in rice

Zhifang Zhao, Aiqing Sun, Wenfeng Shan, Xinhang Zheng, Ying Wang, Lu Bai, Yuchen Xu, Zhuo An, Xiaoyi Wang, Yuanmeng Wang, and Jiangbo Fan

OsRbohI Is the Indispensable NADPH Oxidase for Molecular Patterns Induced Reactive Oxygen Species Production in Rice

Zhifang Zhao[1, 2, 3], Aiqing Sun[1, 2, 3], Wenfeng Shan[1, 2], Xinhang Zheng[1, 2], Ying Wang[1, 2], Yuchen Xu[1, 2], Zhuo An[1, 2], Xiaoyi Wang[1, 2], Yuanmeng Wang[1, 2], Jiangbo Fan[1, 2, *]

1 Shanghai Collaborative Innovation Center of Agri-Seeds, School of Agriculture and Biology, Shanghai Jiao Tong University

2 Joint Center for Single Cell Biology, School of Agriculture and Biology, Shanghai Jiao Tong University

[3] These authors contributed equally.

* Correspondence: Jiangbo Fan (fan.jb@sjtu.edu.cn)

Materials and methods

Plant Materials and Growth Conditions

Mutants of *OsRbohI* (*LOC_Os11g33120), OsRbohB* (*LOC_Os01g25820*) and *OsRbohE* (*LOC_Os01g61880*) in the Zhonghua 11 (ZH11) background were generated using CRISPR/Cas9. The CRISPR targets of them were selected with online tool (http://crispr.mit.edu/). The CRISPR constructs were transformed into rice by service agent (WIMI Biotech) and confirmed by DNA sequencing. T-DNA insertion mutants of *OsRbohI* were obtained from rice T-DNA tagging library (POSTECH) under accession numbers PFG_3A-05869 (*osrbohi-1*) and PFG_1A-24742 (*osrbohi-2*). Mutant *osrbohi-3* is shared by Prof. Fangjie Zhao (Nanjing Agricultural University) as reported (Wang et al., 2023). The mutant lines *osrbohb-1* and *osrbohb-2* are kind gifts from Prof. Yuese Ning (Chinese Academy of Agricultural Sciences). Mutants *osrbohb-3* and *osrbohi-4* are obtained from BIOGLE GeneTech. Rice mutant *osrbohb-4* is a Tos17 insertional line (accession ID: NF5029_0_501_1A) gifted by Prof. Kun-Ming Chen (Northwest A&F University) as reported (Shi et al., 2020). Arabidopsis mutant

atrbohd is shared by Prof. Xiufang Xin (Chinese Academy of Sciences) as reported (Yuan et al., 2021). The *atrbohd* complementary lines *AtD-6* and *AtD-7* are gifted by Yan Liang (Zhejiang University) as reported (Qi et al., 2024). Rice plants were grown in a growth room under the conditions of 12-h day, 30℃, 80% relative humidity (RH) followed by 12-h dark, 28℃, 60% RH. Arabidopsis plants were grown in a growth room at 22 °C with a 16-h-light and 8-h-dark cycle with 65% RH.

Arabidopsis transformation

Full-length CDS of *OsRbohB*/*E*/*I* and *AtRbohD* were inserted downstream the native promoter of *AtRbohD* (2041 bp upstream start codon) and then introduced into *Agrobacterium* strain GV3101. All the Arabidopsis transformation were conducted using floral dip method as previously described (Sun et al., 2022). Hygromycin was used to select transformed lines.

qRT-PCR and RT-PCR

To detect the transcripts of *OsRbohB*, *OsRbohE, OsRbohI* and *AtRbohD* in different mutants and corresponding WT, leaves from 10-day-old seedlings grown on 1/2 MS medium in sterile glass vessel (for rice) or culture dish (for Arabidopsis) were ground in liquid nitrogen. Total RNAs were extracted with TaKaRa MiniBEST Universal RNA Extraction Kit. First-strand cDNA synthesis was conducted with ReverTra Ace qPCR RT Master Mix with gDNA Remover (TOYOBO). For *osrbohi-1* and *osrbohi-2*, RT-PCRs were performed with primers spanning T-DNA insertion with *OsACTIN* as an internal control. For Arabidopsis, *AtActin2* was used as an internal control. Primers used are shown in Supplemental Table S1.

ROS measurement

Detection of PAMP triggered ROS were conducted with a chemiluminescence assay as previously described (Wang et al., 2022). The rice sheaths or Arabidopsis leaf discs were treated with 100 μM flg22 (Sangon Biotech), 20 μg/mL chitin (Sigma), or sterile water in 50 mM Tris-HCl buffer (pH 7.5), containing 10 μM L-012 (FUJIFILM) and 10 μg/mL horseradish peroxidase (Sigma). Luminescence was monitored immediately after the treatment and continuously measured at 1-min intervals for 30-45 minutes with Varioskan Flash multireader (BioTek).

Rice Blast Inoculations and Disease Evaluations

Blast fungal punch inoculation of rice was conducted as previously described (Fan et al., 2018) with modifications. *M. oryzae* (isolate RB22) was cultured on oatmeal agar at 25℃ for 10 days to produce spores. Fresh spores were then collected with distilled H_2O containing 0.5% Tween-20 and adjusted to a concentration of 5×10^5 spores/ml. Seedlings were cultured in big pots (16 plants per pot) for six weeks. The middle part of second leaf (count from top) of each plant was punched and the phenotype was scored at 7~12 dpi. The representative leaves of each line were collected and gDNA were extracted using Cetyltrimethylammonium Bromide (CTAB). The fungal biomass was determined by quantifying the fungal transposon *MoPot2* DNA with quantitative PCR, normalized to rice ubiquitin gene (*LOC_Os03 g13170*) (Kawano et al., 2010). PCR primers are listed in Supplemental Table S1.

Immunoprecipitation (IP) and mass spectrometry assay

IP-MS assay was conducted with rice protoplasts as previously described (Fan et al., 2018). Wild type rice seedlings of 10-14-day old cultured under lights on ½ MS medium were used for protoplast isolation. Briefly, OsRbohB and OsRbohI with HA or GFP label were transformed into rice protoplast by PEG-$CaCl_2$. The resulted protoplasts were treated with 20 μg/ml chitin and then IP with HA or GFP beads for mass spectrometry to identify protein phosphorylation sites.

REFERENCE

Fan, J., Bai, P., Ning, Y., Wang, J., Shi, X., Xiong, Y., Zhang, K., He, F., Zhang, C., Wang, R*., et al.* (2018). The Monocot-Specific Receptor-like Kinase SDS2 Controls Cell Death and Immunity in Rice. Cell Host Microbe *23*, 498-510 e495.

Kawano, Y., Akamatsu, A., Hayashi, K., Housen, Y., Okuda, J., Yao, A., Nakashima, A., Takahashi, H., Yoshida, H., Wong, H.L*., et al.* (2010). Activation of a Rac GTPase by the NLR Family Disease Resistance Protein Pit Plays a Critical Role in Rice Innate Immunity. Cell Host & Microbe *7*, 362-375.

Qi, F., Li, J., Ai, Y., Shangguan, K., Li, P., Lin, F., and Liang, Y. (2024). DGK5β-derived phosphatidic acid regulates ROS production in plant immunity by stabilizing NADPH oxidase. Cell Host & Microbe *32*, 425-440.e427.

Shi, Y., Chang, Y.L., Wu, H.T., Shalmani, A., Liu, W.T., Li, W.Q., Xu, J.W., and Chen, K.M. (2020). OsRbohB-mediated ROS production plays a crucial role in drought stress tolerance of rice. Plant Cell Rep *39*, 1767-1784.

Sun, A., Yin, C., Ma, M., Zhou, Y., Zheng, X., Tu, X., and Fang, Y. (2022). Feedback regulation of auxin signaling through the transcription of H2A.Z and deposition of H2A.Z to SMALL AUXIN UP RNAs in Arabidopsis. New Phytol *236*(5), 1721-1733.

Wang, H.-Q., Zhao, X.-Y., Xuan, W., Wang, P., and Zhao, F.-J. (2023). Rice roots avoid asymmetric heavy metal and salinity stress via an RBOH-ROS-auxin signaling cascade. Molecular Plant *16*, 1678-1694.

Wang, Y., An, Z., Zhao, Z., Li, C., and Fan, J. (2022). Real-Time Detection of Reactive Oxygen Species Production in Immune Response in Rice with a Chemiluminescence Assay. Journal of Visualized Experiments *189*, e64776.

Yuan, M., Jiang, Z., Bi, G., Nomura, K., Liu, M., Wang, Y., Cai, B., Zhou, J.M., He, S.Y., and Xin, X.F. (2021). Pattern-recognition receptors are required for NLR-mediated plant immunity. Nature *592*(7852), 105-109..
